# Fructooligosaccharides-driven metabolism of a defined *Lactobacillus* probiotic consortium modulates hepatic lipid accumulation in a reductionist *in vitro* gut microbiota–liver interaction model

**DOI:** 10.3389/fmicb.2026.1866842

**Published:** 2026-09-09

**Authors:** Margherita Finazzi, Federica Bovio, Matilde Forcella, Marina Lasagni, Paola Fusi, Jessica Zampolli, Patrizia Di Gennaro

**Affiliations:** 1 Department of Biotechnology and Biosciences, University of Milano-Bicocca, Milano, Italy; 2 Department of Earth and Environmental Sciences, University of Milano-Bicocca, Milano, Italy

**Keywords:** fructooligosaccharides, *Lactobacillus*, probiotics, *in vitro* reconstructed human gut microbiota model, gut-liver axis, lipid accumulation, steatosis, short-chain fatty acids

## Abstract

**Introduction:**

Metabolic dysfunction-associated steatotic liver disease may be influenced by human gut microbiota-derived metabolites. Fructooligosaccharides (FOS) and probiotic *Lactobacillus* species have been reported to be associated with metabolic improvements. However, the strain-specific contribution of defined probiotic consortia and the interplay between substrate-driven microbial metabolites and hepatic lipid regulation remain insufficiently characterized. This study aims to investigate whether FOS-driven metabolism of a three-strain *Lactobacillus* consortium (*L. plantarum* PBS067, *L. acidophilus* PBS066, and *L. reuteri* PBS072) modulates hepatocyte lipid accumulation in steatotic HepG2 cells within a reconstructed reductionist *in vitro* human gut microbiota model.

**Methods:**

A previously validated minimal core community, representing metabolic functions of the human gut microbiota, was supplemented with the probiotic consortium and cultured in the presence of FOS with an average degree of polymerization ~10. *Lactobacillus* strain growth and modulation, transcriptional activation of FOS-related genes, and short-chain fatty acid (SCFA) metabolic profiles were characterized. Microbiota-derived metabolites were then tested on palmitate-induced steatotic HepG2 cells.

**Results and discussion:**

FOS supported growth of both probiotic and minimal core members in a strain-dependent manner within the community, with higher growth levels of *L. plantarum* PBS067 and *L. reuteri* PBS072. RT-qPCR analyses of *sacA, scrB*, and *sacK1* genes revealed species-specific transcriptional activation and context-dependent regulation of FOS utilization pathways. Microbial metabolic profiling showed production of SCFAs and organic acids typical of carbohydrate fermentation. Treatment of steatotic HepG2 cells with these metabolites significantly reduced intracellular lipid accumulation and down-regulated Cd36 gene encoding a key fatty acid transporter involved in palmitic acid-induced steatosis. These findings suggest that FOS-driven microbial metabolism within a defined *Lactobacillus* consortium could be associated with hepatocyte lipid uptake under controlled *in vitro* conditions, highlighting the interplay among microbial transcriptional activation and their metabolic profiles.

## 1 Introduction

Metabolic dysfunction-associated steatotic liver disease (MASLD), previously referred to as metabolic dysfunction-associated fatty liver disease (MAFLD) or non-alcoholic fatty liver disease (NAFLD), is identified as a condition marked by hepatic steatosis occurring in the context of metabolic dysregulation, including obesity, type 2 diabetes mellitus (T2DM), or other dysfunctions (Eslam et al., 2020). This condition affects approximately a quarter of the world's adult population (25%−35%), causing a substantial health and economic burden on societies worldwide (Younossi et al., 2018; Eslam et al., 2020). To unravel the complex and multifactorial pathogenesis of MASLD, two models have been proposed (Benedict and Zhang, 2017; Ma et al., 2025). In the classical “two hit” hypothesis, insulin resistance arising from obesity and T2DM promotes hepatic steatosis, whereas a second hit, driven by inflammatory cytokines, lipid peroxidation, mitochondrial dysfunction, and oxidative stress, leads to disease progression (Fang et al., 2018). A second theory, known as “multiple parallel strikes,” hypothesizes that several metabolic, inflammatory, and oxidative factors act in parallel and synergistically contribute to MASLD development (Ma et al., 2025). Within this multifactorial framework, growing evidence indicates that the gut–liver axis is a key regulator of MASLD pathogenesis. Indeed, the liver is anatomically and functionally connected to the intestine through the portal circulation, resulting in continuous exposure to gut-derived nutrients, microbial components, and metabolites. Alterations in gut microbiota composition and intestinal barrier integrity can increase the translocation of microbial products and metabolites to the liver (Tilg et al., 2016).

Dietary factors are key modulators of the human gut microbiota and consequently of the host responses. Among these, prebiotics such as fructo-oligosaccharides (FOS) have attracted considerable attention. Both preclinical and clinical studies indicate that FOS supplementation can attenuate hepatic steatosis and metabolic dysfunction by modulating gut microbiota function and reducing endotoxemia. Moreover, microbial-derived metabolites have been shown to influence hepatic lipid metabolism, insulin sensitivity, and inflammatory response (Delzenne et al., 2011; Bindels et al., 2015; Canfora et al., 2019; Cani et al., 2007; Huang et al., 2023). Thus, FOS-driven microbial metabolism has a prominent role in regulating the gut-liver axis in the context of MASLD. In addition to prebiotics, probiotics can influence the human gut microbiota by modulating microbial metabolic products, strengthening intestinal barrier integrity, and reducing the translocation of pro-inflammatory microbial metabolites to the liver (Sanders et al., 2019). Among probiotic taxa, the genus *Lactobacillus* has been extensively studied for its metabolic versatility and capacity to ferment complex carbohydrates, including FOS. *Lactobacillus* species are known to produce a range of bioactive metabolites that have been associated with improved lipid metabolism, relative to MASLD pathogenesis (Kobyliak et al., 2016; Markowiak and Sliżewska, 2017). Consistently, several experimental and clinical studies have reported that *Lactobacillus*-based probiotic interventions can improve hepatic steatosis and metabolic parameters (Abenavoli et al., 2019; Mularczyk et al., 2021; Huang et al., 2020).

Many *in vivo* studies, particularly in rodents, reflect the physiological complexity of host-microbiota interactions involving prebiotics, individual probiotic strains or their metabolites to investigate how microbial metabolites affect hepatic steatosis and metabolic dysfunction (Cao et al., 2023; Teng et al., 2024; Park et al., 2020). However, these models often limit the ability to disentangle cause-and-effect relationships due to the simultaneous contributions of multiple host, microbial, and environmental variables. More complex *in vitro* gut-mimicking systems, including donor-derived microbiota-based models, dynamic fermentation platforms, and integrated gut-liver approaches, can more closely reproduce human gut physiology and interindividual variability. Nevertheless, reductionist *in vitro* microbial community models remain useful controlled and reproducible frameworks to investigate gut microbiota-host metabolic interactions while preserving key ecological features, including interspecies interactions and substrate-dependent metabolic responses. These models also enable the setup of defined microbial consortia with strain-specific contributions to evaluate how specific dietary substrates, such as prebiotics, shape strain-level microbial dynamics, metabolic outputs resulting from microbial interactions, and their potential influence on host cell function (Bayer et al., 2021; De Roy et al., 2014; Van de Wiele et al., 2015).

The aim of this study is to investigate whether a defined three-strain *Lactobacillus* probiotic consortium (*L. plantarum* PBS067, *L. acidophilus* PBS066, and *L. reuteri* PBS072) can modulate hepatic lipid accumulation and MASLD-related gene expression through microbiota-derived metabolites. To this end, a reductionist reconstructed *in vitro* human gut microbiota (HGM) model, consisting of a previously validated four-strain minimal core community (De Giani et al., 2024; Finazzi et al., 2025), was supplemented with the probiotic formulation and cultured in the presence of fructooligosaccharides with an average degree of polymerization of approximately 10 (FOS DP ~ 10). Within this framework, the influence of FOS availability on microbial dynamics, metabolic outputs, and transcriptional activation of carbohydrate utilization pathways was investigated across the probiotic consortium, the minimal core, and their combined community. Finally, microbiota metabolites derived from FOS fermentation were evaluated for their capacity to modulate lipid accumulation and MASLD-related gene expression in palmitate-induced steatotic HepG2 cells.

## 2 Materials and methods

### 2.1 Bacterial strains and culture conditions

The bacterial strains used in this study are listed in Table 1. Probiotic strains (*Lactiplantibacillus plantarum* PBS067-DSM 24937, *Lactobacillus acidophilus* PBS066-DSM 24936, and *Limosilactobacillus reuteri* PBS072-DSM 25175) were supplied by SynBalance S.r.l (Origgio, Italy) (Presti et al., 2015). Bacterial strains included in the minimal core are *Bacteroides cellulosilyticus, Clostridium symbiosum*, and *Flavonifractor plautii* (sourced from BEI Resources, NIAID, NIH collection, as part of the Human Microbiome Project), and *Escherichia coli* (deriving from the American Type Culture Collection, ATCC, Manassas, VA, USA).

**Table 1:** Bacterial strains included in the human gut reconstructed community used in the study.

Strain	Source	Abbreviation	Role in this study
*Lactiplantibacillus plantarum* PBS067 (DSM 24937)	Human	LP	Probiotic
*Lactobacillus acidophilus* PBS066 (DSM 24936)	Human	LA	Probiotic
*Limosilactobacillus reuteri* PBS072 (DSM 25175)	Human	LR	Probiotic
*Bacteroides cellulosilyticus* CL02T12C19 (HM-726)	Human	BC	Minimal core
*Clostridium symbiosum* WAL-14673 (HM-319)	Human	CS	Minimal core
*Flavonifractor plautii* (previously *Clostridium orbiscindens*) 1_3_50AFAA (HM-303)	Human	FP	Minimal core
*Escherichia coli* ATCC 25922	Human	EC	Minimal core

Probiotic strains were routinely cultured in de Man, Rogosa, and Sharpe (MRS) (Conda Lab, Madrid, Spain) medium supplemented with 0.3% w/v L-cysteine (Merk, Milano, Italy). Minimal core strains were cultured in Reinforced Clostridial Medium (RCM) supplemented with 0.3% w/v L-cysteine and 0.001 % w/v hemin (Hemin chloride, Cayman Chemical, Ann Arbor, MI, USA). All strains were grown statically under anaerobic conditions generated by the Anaerocult GasPack System (Merck, Darmstadt, Germany) at 37 °C.

### 2.2 Growth assays on FOS

Growth assays were performed by cultivating bacteria in 10 mL modified MRS medium (mMRS) supplemented with 0.3% w/v cysteine (De Giani et al., 2022a), and either 2% w/v fructooligosaccharides with an average degree of polymerization of 10 (FOS DP ~ 10) or 1% w/v glucose as the sole carbon source. Initially, bacterial strains were inoculated individually (monoculture) with an initial optical density at 600 nm (OD_600nm_) of 0.1. Subsequently, strains were inoculated at initial OD_600nm_ equal to 0.05 combined to obtain the probiotic consortium (*L. plantarum* PBS067, *L. acidophilus* PBS066, and *L. reuteri* PBS072), the minimal core (*F. plautii, C. symbiosum, B. cellulosilyticus*, and *E. coli*), or the whole community (probiotics and minimal core). All setups were grown statically under anaerobic conditions (Anaerocult GasPack System, Merck, Darmstadt, Germany) at 37 °C for 24 h (only for probiotic consortium) and 48 h. After incubation, OD_600nm_ was measured, and both cells and supernatants were collected for subsequent DNA, RNA, and metabolite analyses.

### 2.3 Total DNA extraction and quantification of bacterial strains by real-time quantitative PCR (RT-qPCR)

Genomic DNA was extracted from monocultures using the GeneJET Genomic DNA Purification Kit (Thermo Fisher Scientific, USA) according to the manufacturer's instructions. For bacterial consortia, including the probiotic formulation, the minimal core, and the whole community, DNA was extracted by using the Stool Nucleic Acid Isolation kit (Norgen Biotek Corp., Thorold, ON, Canada) according to the manufacturer's instructions with minor modifications (De Giani et al., 2022b). Extracted DNA was quantified and assessed for purity using spectrophotometric analysis (NanoDrop One Microvolume UV–Vis Spectrophotometer, ThermoFisher Scientific, Monza, Italy) prior to downstream analysis.

Single-strain modulation within the probiotic consortium, the minimal core, and the whole community was quantified by Real-Time quantitative PCR (RT-qPCR) using PCR Real-Time StepOne Plus (Applied Biosystems, Monza, Italy) and the PowerUp SYBR Green Master mix (Applied Biosystems, Monza, Italy) with species-specific primers (Supplementary Table S1). Absolute quantitation of each bacterial strain was performed using calibration curves from serial dilutions of strain-specific genomic DNA. For each target, standard curves were constructed, and the resulting slope and *y*-intercept values were used to calculate the bacterial concentration expressed as bacterial counts/mL. Quantitative PCR assays were performed using an initial denaturation step at 95 °C for 10 min, followed by 40 cycles of 95 °C for 15 s and 65 °C for 1 min (melt curve: 95 °C for 15 s, followed by 60 °C for 1 min, and 95 °C for 15 s). All DNA samples were analyzed in triplicates.

### 2.4 Gene identification for FOS utilization

Genes potentially involved in FOS utilization were identified by using an *in silico* homology approach. Reference sequences of genes associated with fructan hydrolysis, fructose transport, and intracellular fructose metabolism were retrieved from the literature and from publicly available reference genomes of *Lactobacillus* strains (Barrangou et al., 2006; Saulnier et al., 2007).

Sequence comparisons were performed using the selected reference sequences against the genomes of the three probiotic strains by the RAST (Rapid Annotations using Subsystem Technology) server within the NMPDR platform. Candidate genes were identified based on amino acid sequence similarity and the conserved functional domains of the correspondent gene products. Publicly available databases, including NCBI pipeline (Altschul et al., 1990) and UniProt (The UniProt Consortium, 2025), were used for gene validation.

### 2.5 RNA manipulation and microbial gene expression by real-time quantitative PCR (RT-qPCR)

Total RNA was extracted from 1.5 mL bacterial cultures with OD_600nm_ ~ 3 using the RNeasy PowerSoil Total RNA kit (Qiagen, Milano, Italy) according to the manufacturer's instructions. Extracted DNA was quantified and assessed for purity using spectrophotometric analysis (NanoDrop One Microvolume UV–Vis Spectrophotometer, ThermoFisher Scientific, Monza, Italy) prior to downstream analysis. Complementary DNA (cDNA) was synthesized from 500 ng of total RNA using the iScript cDNA Synthesis Kit (Bio-Rad, Milano, Italy) following the manufacturer's protocol.

Gene expression analysis was performed by quantitative RT-qPCR by using cDNA synthesized. RT-qPCR reactions were carried out using PowerUp SYBR Green Master mix (Applied Biosystems, Monza, Italy) on a PCR Real-Time StepOne Plus (Applied Biosystems, Monza, Italy). Relative gene expression levels were calculated using the 2^(−ΔΔCt)^ method. For each sample, target gene expression was normalized to the 16S rRNA gene, which was used as the internal reference control. The same species-specific 16S rRNA primer sets employed for strain quantification were used for normalization (Supplementary Table S1). Primers targeting genes involved in probiotics' FOS utilization were designed in this study based on the corresponding nucleotide sequence identified in the genomes (Supplementary Table S2). Gene expression levels were normalized to the corresponding glucose-grown condition, which served as the reference control. RT-qPCR assays were performed using the same program described for DNA quantification. All reactions were performed in triplicates.

### 2.6 Microbial metabolite extraction and gas chromatography-mass spectrometry (GC-MS) analysis

Microbial metabolites were extracted from culture supernatants collected after bacterial growth using ethyl acetate at a 1:2 ratio (Merck, Milano, Italy) following the method reported by De Giani et al. (2022a). An aliquot of the extracted organic phase was derivatized with N,O-bis(trimethylsilyl)-trifluoroacetamide (BSTFA) using a BSTFA:sample ratio equal to 1:3 at 60 °C for 20 min. The derivatized samples (1 μL) were qualitatively analyzed using a gas chromatograph (Technologies 6890 N Network GC System) equipped with a mass selective detector (5973 Network, Agilent Technologies, Santa Clara, CA, USA). Separation was performed on a capillary J&W DB-5ms Ultra Inert GC Column (fused silica, 60 m × 0.25 mm, 0.25 μm, Agilent Technologies, Santa Clara, CA, USA). Splitless injection mode was employed, with 99.99% He as the carrier gas (Sapio, Bergamo, Italy), at 70 eV in full scan mode (40–500 Da). The oven was set as follows: 65 °C for 2 min, followed by 8 °C/min to 110 °C, then 17 °C/min to 260 °C, and held at 260 °C for 10 min. The resulting chromatograms were analyzed using MSD ChemStation E.02.02.1431 (Agilent Technologies, Santa Clara, CA, USA). Metabolite identification was achieved by comparison of retention times and mass spectra with those of authentic analytical standards and reference spectra from the National Institute of Standards and Technology (NIST) library, considering only library matches with similarity scores ≥90%.

### 2.7 Cell culture and steatosis induction

The HepG2 human liver cancer cell line (ATCC HB-8065) was grown in RPMI-1640 medium supplemented with heat-inactivated 10% Fetal Bovine Serum (FBS), 2 mM L-glutamine, 100 U/mL of penicillin, and 100 μg/mL of streptomycin and maintained at 37 °C in a humidified 5% CO_2_ incubator. The HepG2 cell line was validated by short tandem repeat profiles that were generated by the simultaneous amplification of multiple short tandem repeat loci and amelogenin (for gender identification).

Steatosis was induced in HepG2 cells by adding 0.25 mM palmitic acid (PA) for 24 h. PA-bovine serum albumin (BSA) conjugate was prepared through the soaping of PA with sodium hydroxide and mixing with BSA. Briefly, a 100 mM solution of PA in 0.1 M NaOH was incubated at 70 °C for 30 min, diluted 1:20 with 10% BSA, vortexed for 30 s, and incubated further for 15 min at 55 °C, obtaining a working solution of 5 mM PA.

Reagents for cell cultures were supplied by EuroClone (EuroClone S.p.A, Milan, Italy) and chemicals by Merck (Merck, Darmstadt, Germany).

### 2.8 Cell viability assay

For the evaluation of cell viability, cells were seeded in 96-well microtiter plates at a density of 1 × 10^4^ cells/well, and 24 h later, treated with 0.1 and 0.5 mg/mL of each extract. After 48 h, cell viability was assessed using an *in vitro* MTT-based toxicology assay kit according to the manufacturer's protocols (Merck, Darmstadt, Germany). The absorbance was measured at 570 nm using a Spectrostar Nano Microplate Reader (BMG LABTECH, Ortenberg, Germany). Results were expressed as mean values ± standard error of at least three independent experiments.

### 2.9 Oil Red *O* staining of HepG2 cells

To determine intracellular lipid accumulation, Oil Red *O* staining was performed. HepG2 cells were seeded in 24-well plates at a density of 2.5 × 10^4^ cells/well or grown on cover slips in 6-well plates at a density of 1.25 × 10^5^ cells/well, and 24 h later, treated with 0.1 mg/mL for each extract. After 24 h, treatment was removed, and HepG2 cells were stressed with 0.25 mM PA for an additional 24 h to induce steatosis. After treatment, the cells were carefully washed twice with Phosphate Buffer Solution (PBS) and fixed with 4% paraformaldehyde for 15 min at room temperature. After fixation, cells were washed twice with PBS, stained with 3 mg/mL Oil Red *O* in 60% aqueous isopropanol for 20 min at room temperature, and then rinsed with PBS. Images were acquired with a Thunder microscope (Leica Microsystems, Milano, Italy) and light microscopy at 10 × magnification, using a Zeiss Axioplan microscope equipped with a digital camera and analyzed with ImageJ software (NIH), and the mean red intensity was quantified and normalized on total cells.

### 2.10 HepG2 gene expression analysis by RT-qPCR

HepG2 cells were seeded in 6-well plates at a density of 2 × 10^5^ cells/well, and 24 h later, treated with 0.1 mg/mL of each extract. After 24 h, treatment was removed, and HepG2 cells were stressed with 0.25 mM PA for further 24 h to induce steatosis. The total RNA was isolated using Quick-RNA TM MiniPrep (Zymo Research, Irvine, CA, USA), according to the manufacturer's instructions. Total RNA was reverse-transcribed using SuperScript II Reverse Transcriptase (Invitrogen, Carlsbad, CA, USA), oligo dT and random primers, according to the manufacturer's protocol. For quantitative real-time PCR (qPCR), SYBR Green method was used. Briefly, 50 ng cDNA was PCR amplified with Luna Universal qPCR Master Mix (New England BioLabs, Hitchin, Hertfordshire, UK) and specific primers, using an initial denaturation step at 95 °C for 10 min, followed by 40 cycles of 95 °C for 15 s and 59 °C annealing for 1 min. Each sample was normalized using the *Pol2* gene as an internal reference control. The relative expression level was calculated with the 2^(−Δ*ΔCt*)^ method and was expressed as a relative fold change between treated and untreated cells (Livak and Schmittgen, 2001). The primers used for qPCR are reported in Supplementary Table S3.

### 2.11 Statistical analysis

Data derived from bacterial growth assays are presented as mean values ± standard error. Statistical significance was assessed using Student's *t*-test and defined as *p*-value < 0.05 (^*^), < 0.01 (^**^), or < 0.001 (^***^).

Bacterial counts/mL obtained by RT-qPCR analysis are reported as mean values ± standard error. Statistical significance was evaluated using the Wilcoxon signed-rank test and defined as *p*-value < 0.05 (^*^), or < 0.01 (^**^).

Data derived from HepG2 cell assays, including lipid accumulation and gene expression analyses under palmitic acid-induced steatosis, are presented as mean values ± standard error. All the experiments were carried out at least in biological triplicate. Statistical analyses have been performed with one-way ANOVA followed by a Dunnett's *post hoc* test to evaluate the effect compared to FOS 0.1 mg/ml (indicated by ^*^) and one-way ANOVA followed by a Dunnett's *post hoc* test to evaluate the effect compared to PA 0.25 mM (indicated by #).

Results were considered statistically significant at *p*-value < 0.05 (^*^), < 0.01 (^**^), < 0.001 (^***^), or < 0.0001 (^****^).

## 3 Results

### 3.1 Single-strain growth on FOS DP ~ 10

**Figure 1:**
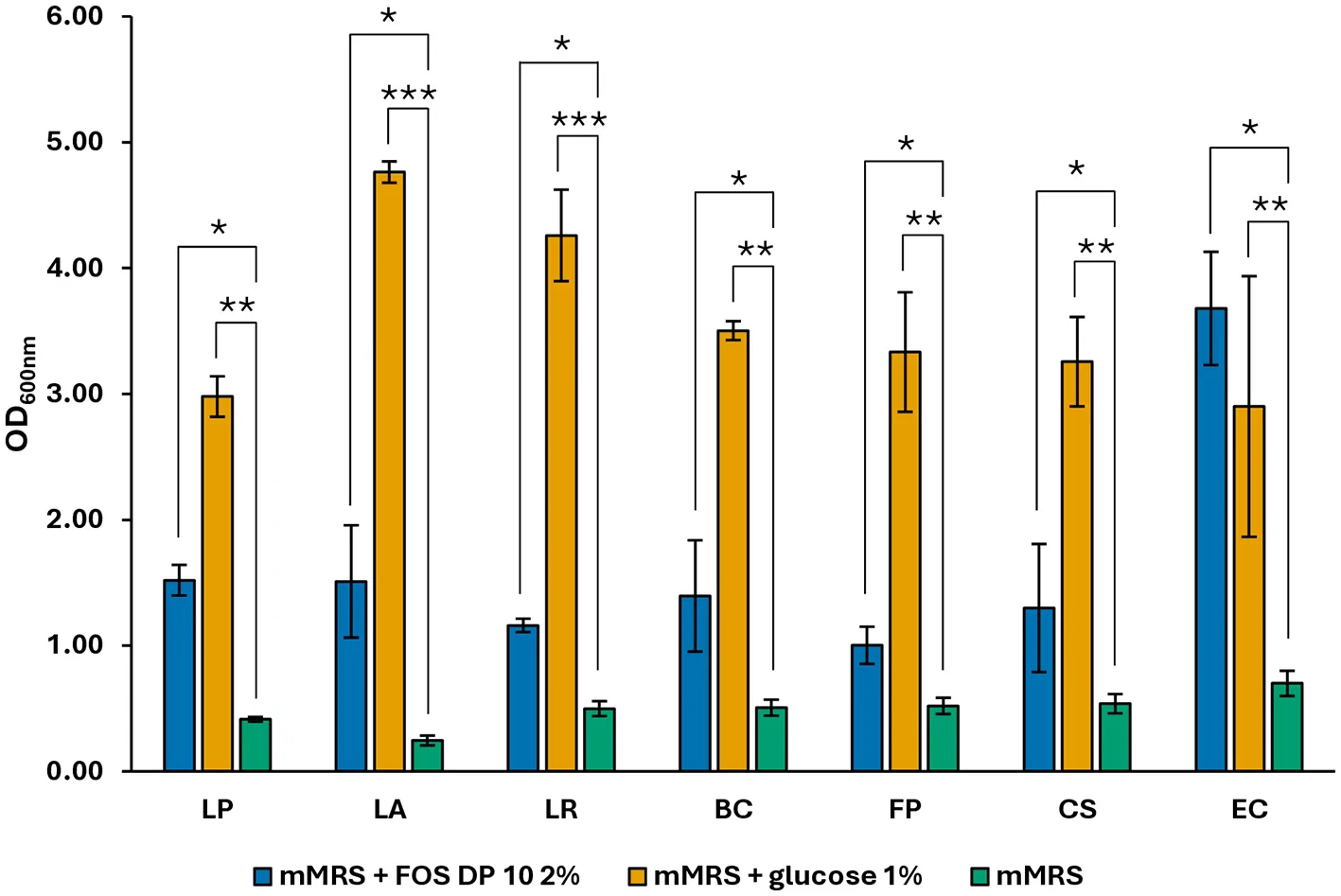
Growth of the single strains on mineral medium mMRS supplemented with FOS DP ~ 10, with glucose (as reference medium). The growth is presented as the mean value of OD_600nm_ ± standard error. The statistical significance was calculated via Student's *t* test: *p-value < 0.05, **p < 0.01, ***p < 0.001.

The ability of individual probiotic and minimal core strains to utilize fructooligosaccharides with an average degree of polymerization of approximately 10 (FOS DP ~ 10) as a carbon source was assessed through growth assays in the presence of FOS DP ~ 10 at a final concentration of 2% w/v or glucose at a final concentration of 1% w/v (reference condition; Figure 1). All three *Lactobacillus* strains, *L. plantarum* PBS067 (LP), *L. acidophilus* PBS066 (LA), and *L. reuteri* PBS072 (LR), were able to grow in the presence of FOS DP ~ 10 (*p*-value < 0.05 vs. mMRS). LP and LA strains displayed the highest growth performance, reaching OD_600nm_ values around 1.5, while LR showed a more moderate but significant growth (OD_600nm_ 1.2). Similarly, strains constituting the minimal core microbiota, *F. plautii* (FP), *C. symbiosum* (CS), *B. cellulosilyticus* (BC), and *E. coli* (EC), were able to grow on FOS DP ~ 10 (for FP, CS, and BC, *p*-value < 0.05 vs. mMRS and for EC, *p*-value < 0.01 vs. mMRS; Figure 1). *E. coli* exhibited the highest growth (OD_600nm_ around 3.5), consistent with its metabolic versatility and growth rate in carbohydrate-rich media, in contrast, the other strains showed lower but significant growth. All the strains were also able to grow in the presence of glucose (used as a reference condition), showing OD_600nm_ values between ~ 3 and 5 (Figure 1).

Overall, these results indicate that FOS DP ~ 10 supports the growth of both probiotic and minimal core members, while revealing clear strain-specific differences in growth efficiency.

### 3.2 Modulation of the *in vitro* reconstructed human gut microbiota in the presence of FOS DP ~ 10

To investigate how substrate availability and microbial dynamics influence the relative abundance of single-strains, strain-specific quantification was performed on DNA extracted after growth on FOS DP ~ 10 growth or glucose (reference condition) after 48 h by RT-qPCR. The bacterial dynamics were assessed on the probiotic consortium alone, the minimal core, and the whole community (composed of the minimal core and the probiotic consortium).

**Figure 2:**
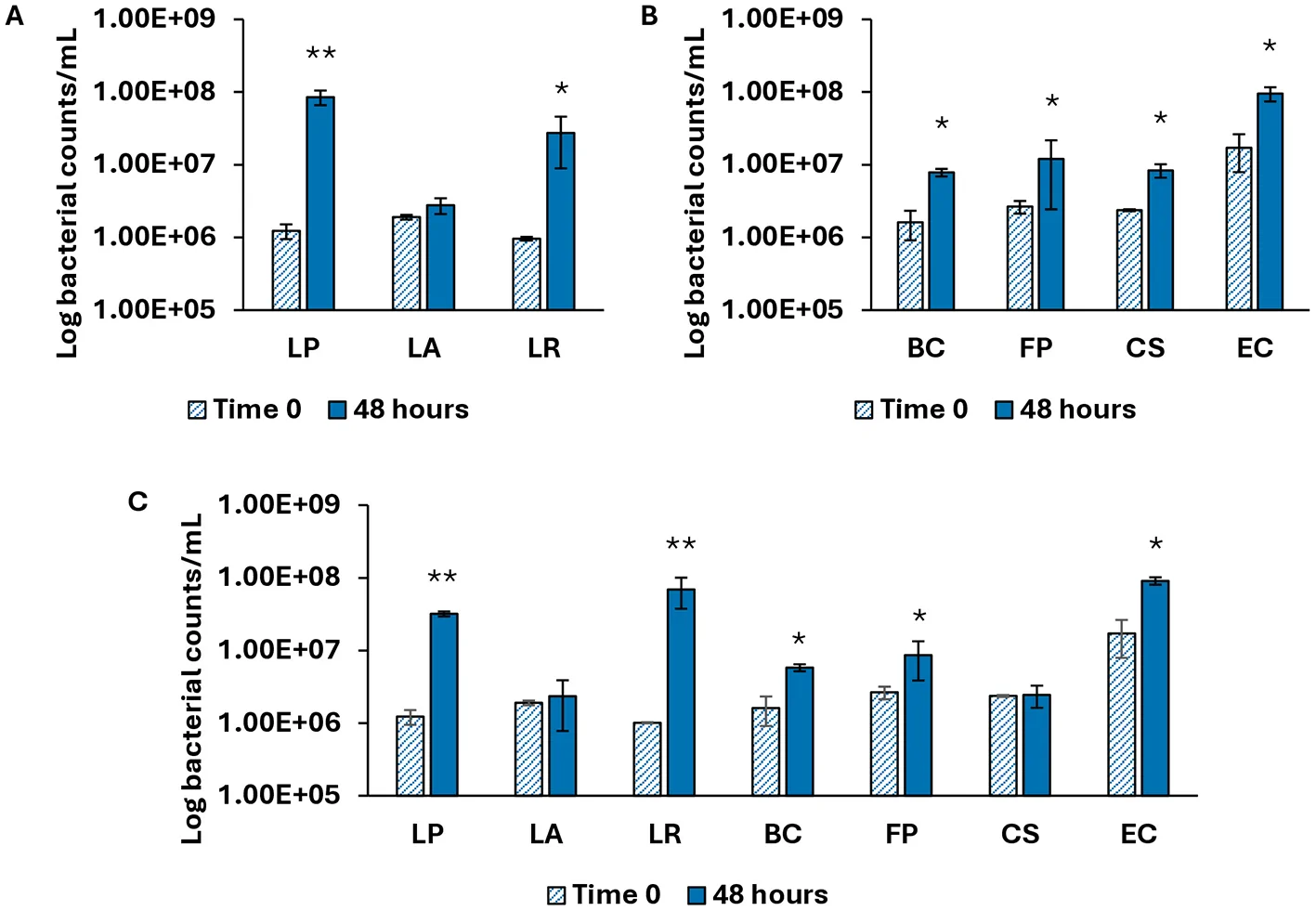
Modulation of the single strains through RT-qPCR. Values of bacterial counts/mL reached by **(A)** the probiotics, **(B)** the minimal core, and **(C)** the whole community during growth on 2% FOS DP ~ 10. Statistical significance was assessed using the Wilcoxon signed-rank test: *p-value < 0.05; **p-value < 0.01.

In the probiotic consortium grown on FOS DP ~ 10, *L. plantarum* PBS067 is the predominant strain at 48 h, reaching ~10^8^ bacterial counts/mL, followed by *L. reuteri* PBS072 (~ 10^7^ bacterial counts/mL; Figure 2A). In the minimal core, all the strains significantly grow in the presence of FOS DP ~ 10, with *E. coli* reaching the highest value of bacterial counts/mL (~10^8^) at 48 h (Figure 2B). The addition of the probiotic consortium to the minimal core further modulated strain-level dynamics. Indeed, in the whole community, FOS promotes the growth of *L. plantarum* PBS067, *L. reuteri* PBS072, and *E. coli* reaching values of bacterial counts around ~10^8^ at 48 h. *B. cellulosilyticus* and *F. plautii* showed values of ~10^7^ bacterial counts/mL (Figure 2C).

**Figure 3:**
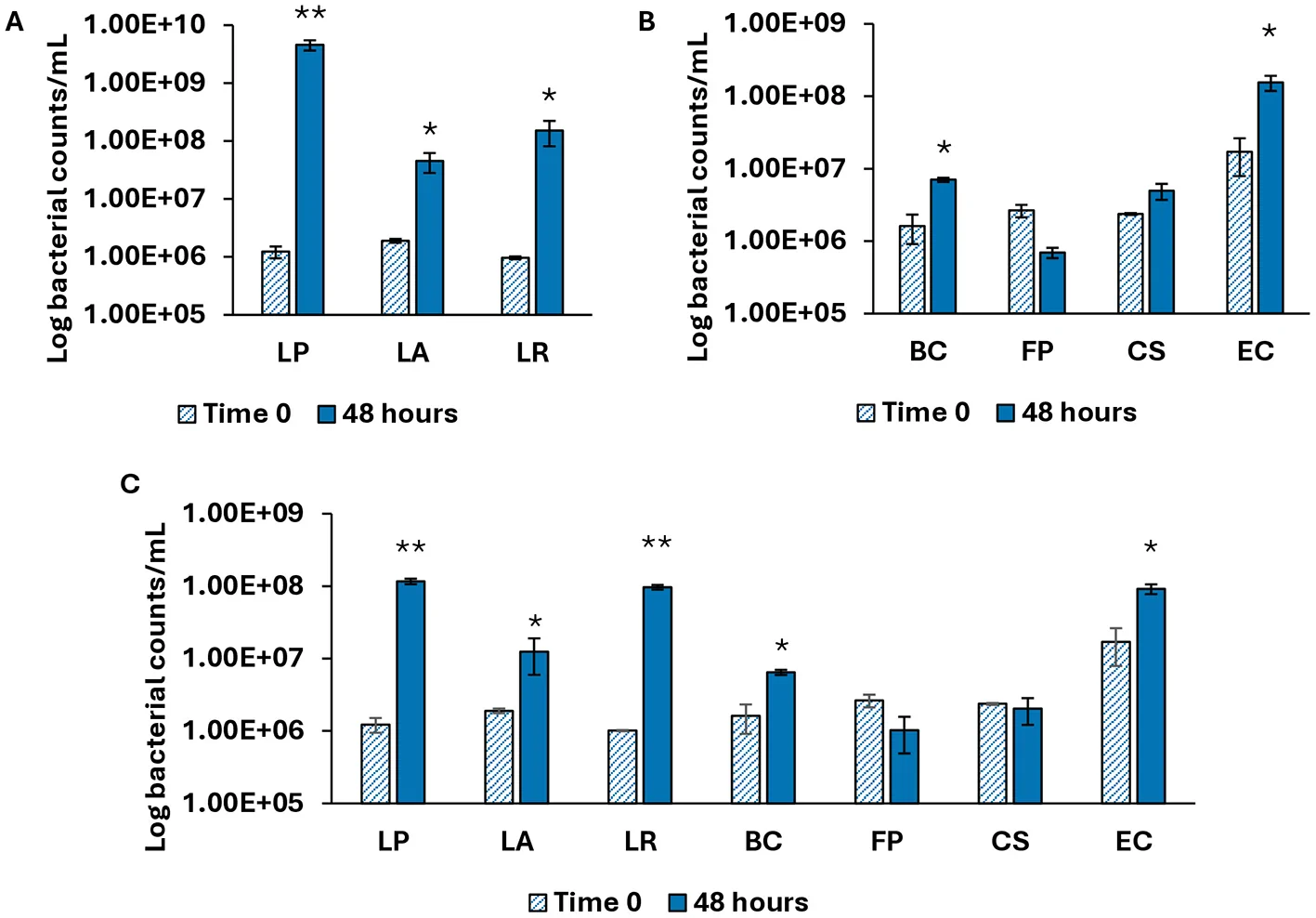
Modulation of the single strains through RT-qPCR. Values of bacterial counts/mL reached by the **(A)** probiotics, **(B)** the minimal core, and **(C)** the whole community during growth on 1% glucose. Statistical significance was assessed using the Wilcoxon signed-rank test: *p-value < 0.05; **p-value < 0.01.

As expected, glucose as a readily fermentable reference substrate, supported the growth of all bacterial strains in the three tested condition as reported in Figure 3.

Overall, these data highlighted that FOS supplied as a prebiotic supported strain-dependent modulation of the probiotic consortium, which is amplified under community conditions.

### 3.3 Identification of genes potentially involved in FOS DP ~ 10 by *Lactobacillus* strains

To investigate the genetic basis of FOS DP ~ 10 utilization by the probiotic strains, a targeted homology-gene identification approach was performed using reference sequences previously described for FOS and sucrose metabolism in *Lactobacillus* sp. Comparative genomic analysis enabled the identification of genes putatively involved in FOS uptake and intracellular catabolism across the three strains included in the probiotic formulation.

**Figure 4:**
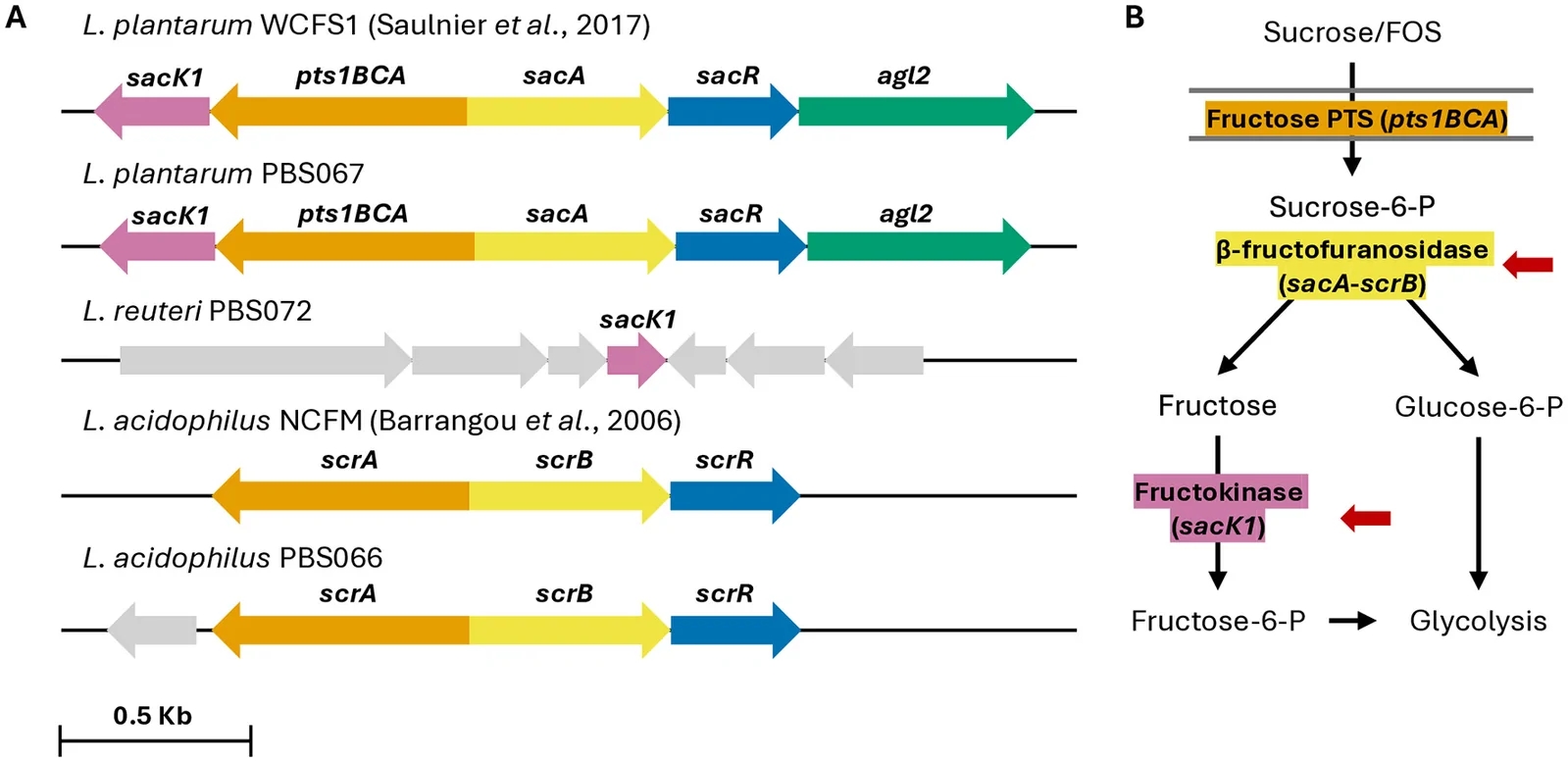
**(A)** Comparison of genes identified in probiotic strains (*L. plantarum* PBS067, *L. reuteri* PBS072, and *L. acidophilus* PBS066) putatively involved in sucrose/FOS utilization with reference strains. Genes encoding fructose/sucrose phosphotransferase system (PTS) components (*pts1BCA* or *scrA*), β-fructofuranosidase (*sacA* or *scrB*), transcriptional regulators (*sacR* or *scrR*), fructokinase (*sack1*), and related enzymes are highlighted. **(B)** Schematic representation of the predicted sucrose/FOS metabolic pathway in *Lactobacillus* spp. illustrating the uptake of sucrose/FOS, intracellular hydrolysis into fructose and glucose-6-phosphate, and subsequent entry into central carbon metabolism. Enzymatic steps associated with the genes identified in panel **(A)** are indicated with the same color. Red arrows indicate genes selected for expression analysis.

In *L. plantarum* PBS067, putative genes were identified through comparative genomic analysis with the reference strain, *L*. *plantarum* WCFS1 (Saulnier et al., 2007) (Figure 4A), consistent with previously described sucrose/FOS utilization pathways. The genes comprise a fructose-specific phosphotransferase system (PTS, *pts1BCA*), a β-fructofuranosidase (*sacA*), a transcriptional regulator (*sacR*), and a fructokinase (*sacK1*).

In *L. reuteri* PBS072, a fructokinase-encoding gene was identified, whereas other components of the canonical FOS utilization operon were not detected (Supplementary Table S4).

By comparing the reference strain, *L*. *acidophilus* NCFM (Barrangou et al., 2006) with *L. acidophilus* PBS066, homologous genes encoding components of the sucrose/fructose utilization system were identified. These genes comprise a fructose-specific phosphotransferase system (*scrA*), a β-fructofuranosidase (*scrB*), and a transcriptional regulator (*scrR*; Figure 4A, Supplementary Table S4).

A schematic overview of the predicted sucrose/FOS metabolic pathway and the enzymatic steps associated with the identified genes is shown in Figure 4B.

Collectively, these data demonstrate species-specific differences in the genetic architecture associated with FOS utilization pathways among the probiotic strains. Based on these findings, key genes involved in FOS uptake and metabolism were selected for subsequent expression analysis under different growth conditions and levels of microbial complexity.

### 3.4 Transcriptional analysis of FOS utilization genes in *Lactobacillus* strains

**Figure 5:**
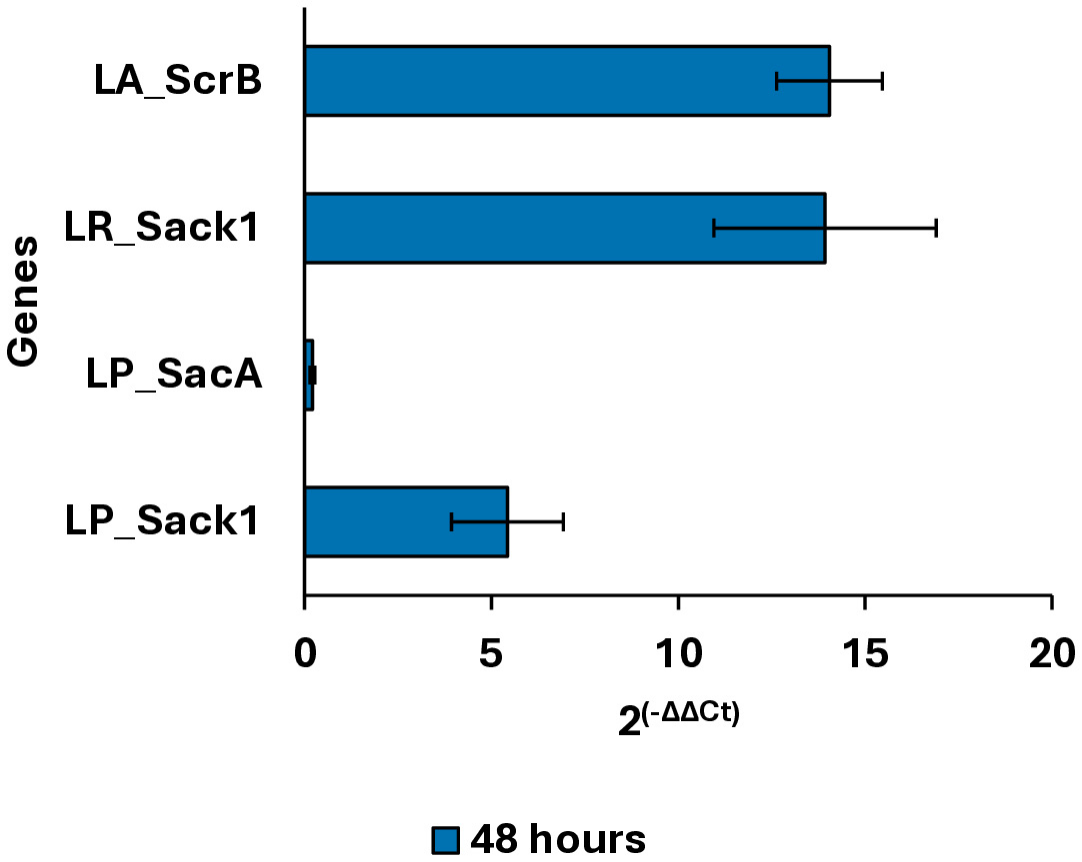
Expression of genes involved in sucrose/FOS metabolism in *L. plantarum* PBS067, *L. reuteri* PBS072, and *L. acidophilus* PBS066 upon growth on 2% FOS DP ~ 10 at 48 h. Gene expression was assessed by RT-qPCR and normalized to strain-specific 16S rRNA reference genes. Data are reported as 2^(−ΔΔCt)^ ± standard deviation.

**Figure 6:**
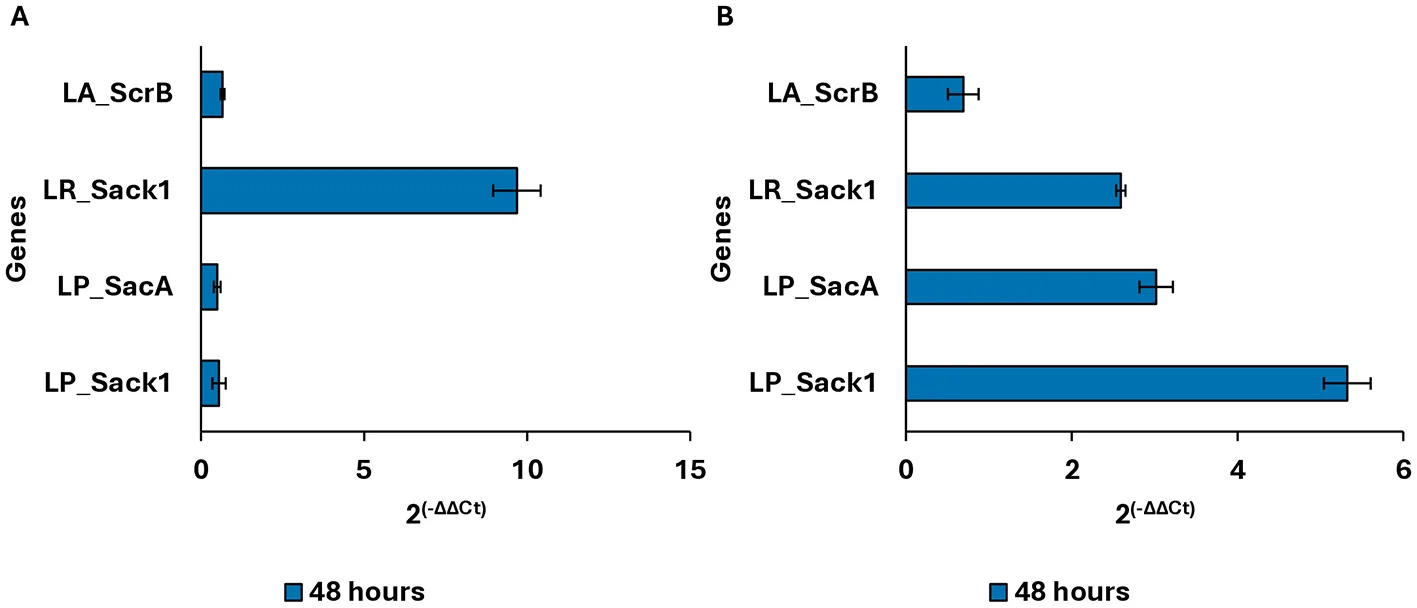
Expression of probiotic genes involved in sucrose/FOS metabolism in **(A)** the probiotic consortium, and **(B)** the reconstructed whole community upon growth on 2% FOS DP ~ 10 at 48 h. Gene expression was assessed by RT-qPCR and normalized to strain-specific 16S rRNA reference genes. Data are reported as 2^(−ΔΔCt)^ ± standard deviation.

The expression of selected genes was analyzed by RT-qPCR in *L. plantarum* PBS067, *L. acidophilus* PBS066, and *L. reuteri* PBS072 grown as single-strain cultures (Figure 5), within the probiotic consortium (Figure 6A), and in the whole community in the presence of FOS (DP ~ 10), compared with glucose-grown condition at 48 h (Figure 6B).

Considering the monocultures, LP displayed a transcriptional activation of the β-fructokinase gene *sacK1* (Figure 5). In contrast, the expression of the fructofuranosidase gene *sacA* was not detected. However, additional analyses revealed that both genes (*sacA* and *sacK1*) are expressed at 24 h (Supplementary Figure S1), suggesting a temporal regulation of the pathway.

In LA and LR, the expression of respectively *scrB* and *sacK1* was detected at 48 h during growth on FOS (Figure 5).

In the probiotic consortium, *sacA* and *sacK1* from LP and *scrB* from LA (Figure 6A) were not expressed at 48 h. In contrast, *sacK1* from LR maintained expression levels comparable to those observed in single-strain culture (Figure 6A). Interestingly, the differential expression of LP genes (*sacA* and *sacK1*) between the monoculture and the probiotic consortium at 48 h suggests that interspecies interactions modulate the transcriptional regulation of FOS utilization pathways. Notably, both *sacA* and *sacK1* were expressed at 24 h within the consortium (Supplementary Figure S2), indicating a shift in temporal expression dynamics.

In the reconstructed microbial community at 48 h, all FOS-related genes identified in the probiotic strains were expressed, with the exception of *scrB* from LA (Figure 6B). Consistent with growth data, the absence of *scrB* expression likely reflects the limited growth of LA under these conditions (Figure 2A). In contrast, genes from LP and LR were expressed, indicating their contribution to FOS-related carbohydrate metabolism wthin the broader microbial consortium.

Overall, these results demonstrate that the transcriptional activation of FOS-related metabolic pathway is strongly influenced by microbial context, and time, with gene expression patterns differing between monoculture, probiotic consortium, and the reconstructed microbial community, rather than being constitutively driven by gene presence alone.

### 3.5 Production of bacterial metabolites and SCFAs

**Figure 7:**
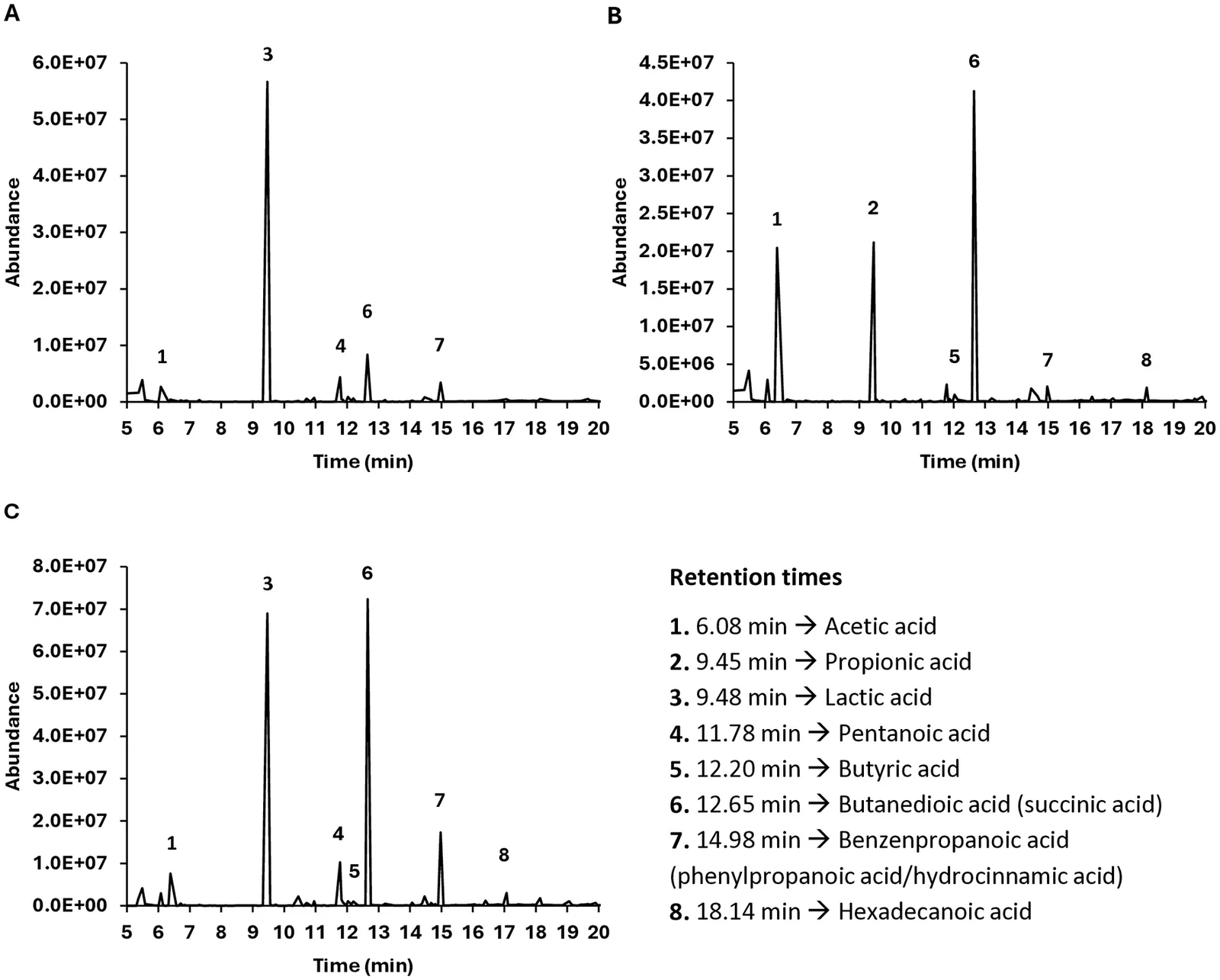
Chromatogram profiles of bacterial metabolites by GC-MSD after 2% FOS DP ~ 10 fermentation. Metabolites produced by **(A)** probiotic consortium, **(B)** minimal core, and **(C)** the whole community.

To characterize the metabolic profiles associated with FOS utilization, metabolites produced by the probiotic formulation, the minimal core, and the reconstructed microbial community were analyzed by gas chromatography-mass spectrometry (GC-MS; Figure 7).

In the probiotic formulation, the metabolic profile showed typical fermentation products of lactobacilli, including acetic acid (peak 1, Rt 6.08), lactic acid (peak 3, Rt 9.48 min), and succinic acid (peak 6, Rt 12.65 min; Figure 7A). The minimal core displayed a distinct metabolite spectrum that included propanoic acid (peak 2, Rt. 9.45 min), butyric acid (peak 5, Rt 12.20 min), and succinic acid (peak 6, Rt. 12.65; Figure 7B). The whole reconstructed microbial community exhibited a broader metabolite profile with a greater number and diversity of detected compounds (Figure 7C).

In addition, metabolite profiling was also performed on cultures grown on glucose (reference condition; Supplementary Figure S3). Overall, glucose-grown cultures displayed metabolite profiles quite similar to those observed on FOS.

### 3.6 Effects on HepG2 cells with palmitic acid-induced steatosis

**Figure 8:**
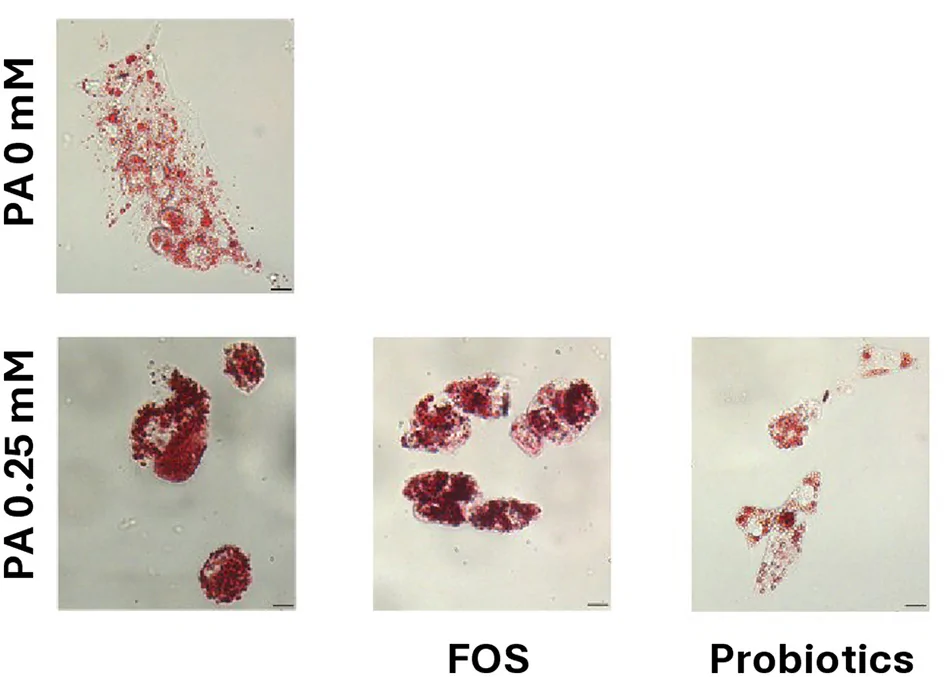
Images of not-treated and 24 h-pre-treated HepG2 cells using 0.1 mg/mL of bacterial metabolites derived from probiotic consortium grown on 2% FOS DP ~ 10 and then exposed to 0.25 mM PA for further 24 h for steatosis induction. Images were taken with Thunder microscope (Leica Microsystems) at 40 × magnification. Scale bar: 10 μm.

**Figure 9:**
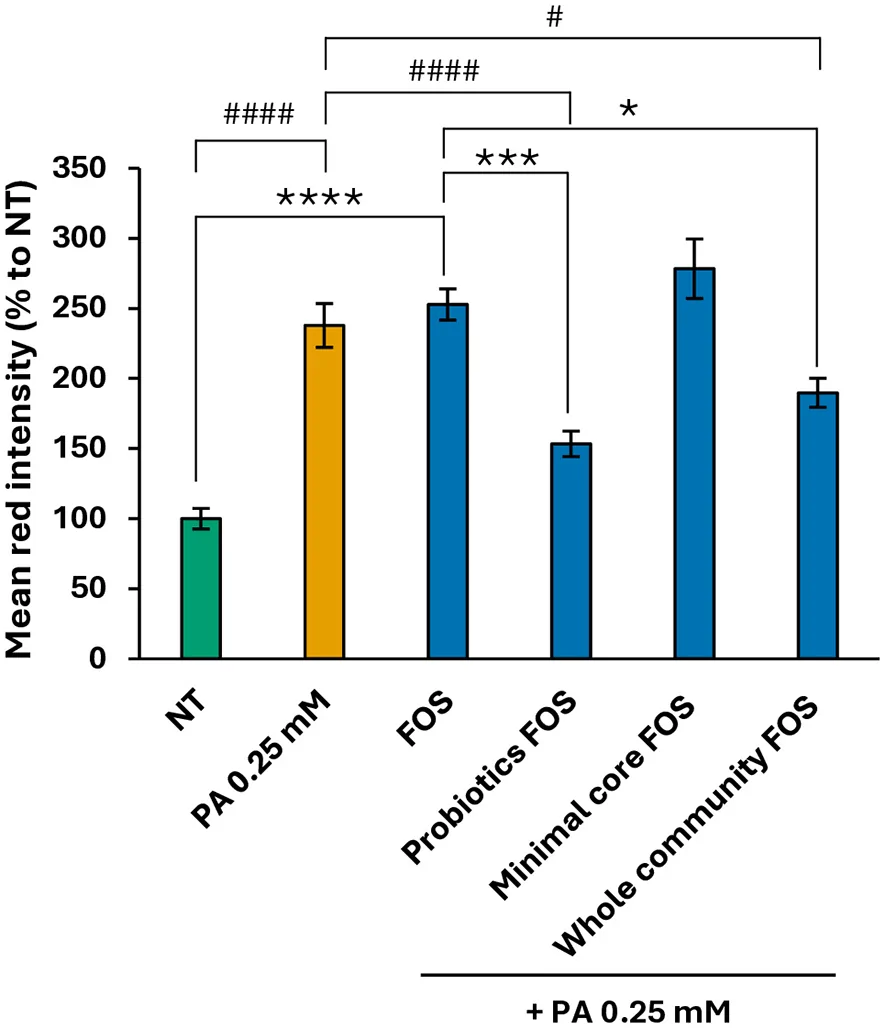
Effect of the extract of bacterial metabolites on HepG2 cells with palmitic acid-induced steatosis. The figure shows lipid accumulation in HepG2 cells treated for 24 h with 0.1 mg/mL of bacterial metabolites derived from probiotic consortium, minimal core, and whole community grown on 2% FOS DP ~ 10 and then exposed to 0.25 mM PA for further 24 h for steatosis induction. Values are represented as mean red intensity percentage ± standard error. Statistical analyses have been performed with one-way ANOVA followed by a Dunnett's *post hoc* test to evaluate the effect compared to FOS 0.1 mg/ml (indicated by *) and one-way ANOVA followed by a Dunnett's *post hoc* test to evaluate the effect compared to PA 0.25 mM (indicated by #). Statistical significances were at *p*-value < 0.05 (*), < 0.01 (**), < 0.001 (***), or < 0.0001 (****).

**Figure 10:**
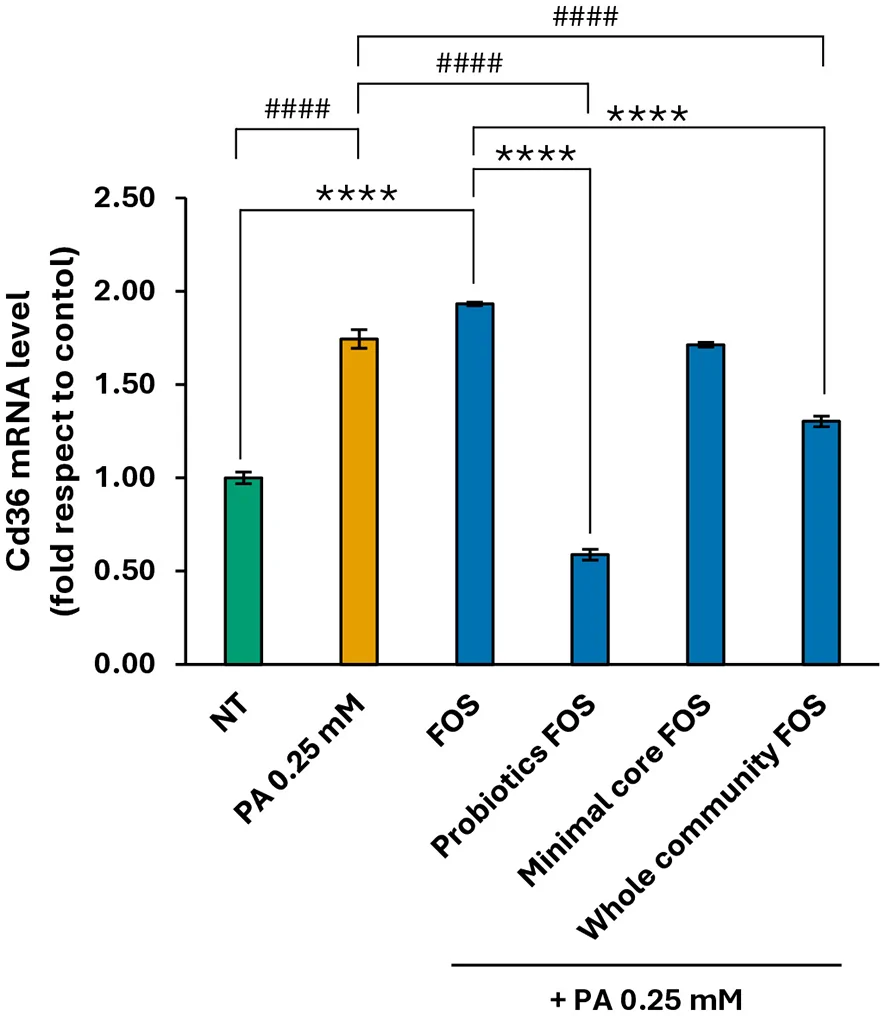
Effect of the extract of bacterial metabolites on HepG2 cells with palmitic acid-induced steatosis. The figure shows *Cd36* gene expression levels in HepG2 cells treated for 24 h with 0.1 mg/mL of bacterial metabolites derived from probiotic consortium, minimal core, and whole community grown on 2% FOS DP ~ 10 and then exposed to 0.25 mM PA for further 24 h for steatosis induction. Values are represented as mean fold respect to control ± standard error. Statistical analyses have been performed with one-way ANOVA followed by a Dunnett's *post hoc* test to evaluate the effect compared to FOS 0.1 mg/ml (indicated by *) and one-way ANOVA followed by a Dunnett's *post hoc* test to evaluate the effect compared to PA 0.25 mM (indicated by #). Statistical significances were at *p*-value < 0.05 (*), < 0.01 (**), < 0.001 (***), or < 0.0001 (****).

The human hepatocarcinoma HepG2 cell line exposed to palmitic acid (PA) can be used as an *in vitro* model of metabolic dysfunction-associated steatotic liver disease (MASLD) (Ramos et al., 2022). A 24-h treatment with 0.25 mM PA is sufficient to cause a significant accumulation of lipid droplets in the cells, as shown by the specific Oil Red *O* staining for lipid droplets, without reducing more than 50% cell viability, thus allowing the real effect of the samples to be evaluated while minimizing confounding effects due to cell death mechanisms (Supplementary Figure S4). To investigate whether the extract of bacterial metabolites could protect hepatic cells from steatosis, HepG2 cells were firstly pre-treated for 24 h with bacterial metabolites derived from probiotic consortium, minimal core, and whole community grown on 2% FOS DP ~ 10 and then exposed to 0.25 mM PA for further 24 h for steatosis induction. Cells pre-treated with 0.1 mg/mL FOS DP ~ 10 or bacterial metabolites derived from minimal core showed the same lipid accumulation of steatotic cells; while a significantly lower intracellular lipid accumulation was found in cells pre-treated with 0.1 mg/mL bacterial metabolites derived from probiotic consortium and whole community compared to both steatotic HepG2 cells and FOS DP ~ 10 (Figures 8, 9). Subsequently, to understand if this reduction in lipid droplets was due to changes in fatty acid metabolism, we evaluated the expression levels of *SREBP* and *PPAR*α, two key lipid metabolism regulators, *ACC, Fasn* and *HMGCR*, coding for the anabolic enzymes acetyl-CoA carboxylase, fatty acid synthase and 3-hydroxy-3-methylglutaryl-CoA reductase, respectively, and *Cd36*, a critical fatty acids sensor and lipid transporter inside the cell. Steatotic cells showed a significantly higher mRNA level of *Cd36* compared to untreated cells, with no changes in the other genes analyzed (Supplementary Figure S4D). The expression level of this gene was also investigated in cells pre-treated with bacterial metabolites. HepG2 cells pre-treated with 0.1 mg/mL bacterial metabolites derived from probiotic formulation and whole community showed a reduction of *Cd36* mRNA level compared to steatotic cells and cells pre-treated with FOS DP ~ 10, or bacterial metabolites derived from minimal core cells in a trend similar to the one of intracellular lipid content (Figure 10).

## 4 Discussion

The present study suggests that the modulation of FOS-driven microbial metabolism by a *Lactobacillus* probiotic formulation influences the hepatocyte lipid accumulation within a simplified and defined human gut microbiota-host *in vitro* framework.

This reductionistic system was applied to a seven-species microbial community comprising a three *Lactobacillus* species representing the probiotic consortium and four microbial species selected as representatives of the commensal core, to better evaluate the functional groups of the gut microbiota. By integrating strain-level dynamics, transcriptional regulation, metabolic profiling, functional effects on hepatocytes, and MASLD-related gene expression, the present multi-layered study shows how the probiotic-driven microbial activity may shape host lipid metabolism.

Growth analyses demonstrated that FOS DP ~ 10 supports cell growth of both probiotic strains and minimal core members, although with strain-dependent efficiency. Across microbial setups, FOS promoted a differentiation among probiotic *Lactobacillus* strains, with higher bacterial cell levels of *L*. *plantarum* PBS067 and *L*. *reuteri* PBS072. This finding suggests that substrate complexity can modulate the microbial community and the strain specific responses, in agreement with previous reports describing the ability of lactobacilli to metabolize medium-chain FOS (Li et al., 2015; Yao et al., 2022).

Transcriptional analysis revealed probiotic species-specific differences in the gene expression for FOS utilization pathways, supporting the ability of these microorganisms to utilize this carbon source. This is in line with literature reporting that *Lactobacillus* FOS metabolism is often encoded by genes reflecting ecological adaptation and niche-specific carbohydrate utilization strategies (Saulnier et al., 2007; Chen et al., 2015; Cui et al., 2021; Barrangou et al., 2003). The identification of genes involved in FOS metabolism in *L*. *plantarum* is consistent with its higher growth performances. On the other hand, *L. acidophilus* and *L. reuteri* were not preponderant in FOS DP ~ 10 utilization which is often mirrored by gene variable profiles depending on the carbon source (Huang et al., 2021; Andersen et al., 2012; Frese et al., 2011; Spinler et al., 2014). The partial FOS-related genetic repertoire identified in *L*. *reuteri* reflects the evolutionary specialization previously described for this species (Frese et al., 2011; Spinler et al., 2014). The modulation of *sacA, scrB*, and *sacK1* expression across monoculture, probiotic consortium, and the whole community conditions indicates that interspecies interactions influence carbohydrate metabolic activity, likely through competition and metabolic cross-feeding.

Metabolic assessment demonstrated that FOS fermentation produced metabolite profiles typical of carbohydrate fermentation, including short-chain fatty acids (SCFAs) and organic acids such as lactic, propanoic, butyric, and succinic acid. These results align with previous reports indicating that microbial fermentation of different carbohydrates can converge toward common SCFAs and organic acids that can exert systemic metabolic effects (Louis and Flint, 2017; Morrison and Preston, 2016). Interestingly, the reconstructed community exhibited a broader metabolite diversity, reflecting increased metabolic interactions within the *Lactobacillus* consortium which may represent a configuration associated with enhanced microbial metabolic interplay, although its relevance to *in vivo* gut health requires further investigation.

Although it is known that FOS, as a prebiotic supplementation, is generally associated with promoting SCFA production *in vivo* system (De Giani et al., 2022b), the SCFA profiles observed under FOS and glucose conditions in the present study likely reflect intrinsic methodological constraint of the *in vitro* batch fermentation system. In our experimental setup, both substrates are directly accessible to the human reconstructed microbial community, and no host-mediated absorption or spatial compartmentalization occurs. Unlike *in vivo* conditions, where glucose is rapidly absorbed in the upper gastrointestinal tract and does not typically reach the colon in significant amounts (Gray, 1970), *in vitro* systems allow glucose to remain available for microbial fermentation throughout the incubation period. Therefore, in our study, glucose was used only as a fermentable reference carbon source, whereas FOS was included as a prebiotic substrate to assess its potential to selectively support probiotic growth and metabolic activity under conditions that may better advise future *in vivo* applications. While the reductionist *in vitro* model effectively captures the reconstructed microbial metabolic potential, it may not reproduce physiological dynamics of substrate availability, and host-microbiota interactions, underestimating substrate-specific effects that are driven by host physiology *in vivo*.

Microbial-derived metabolites are known to influence host lipid metabolism through multiple mechanisms. SCFAs, in particular, have been shown to regulate hepatic lipid homeostasis by modulating AMPK activation, inhibiting lipogenesis pathways such as SREBP-1c signaling, and improving insulin sensitivity. These mechanisms collectively reduce hepatic lipid accumulation and improve metabolic homeostasis (Xavier-Santos et al., 2020; Torres et al., 2019). In line with these mechanisms, pretreatment of palmitate-stressed HepG2 cells with bacterial metabolites derived from probiotic fermentation resulted in reduced intracellular lipid accumulation. This supports the hypothesis that microbial metabolic products may contribute to changes in hepatocyte lipid handling. Importantly, metabolite treatment also reduced expression of *Cd36*, a key fatty acid transporter involved in hepatic lipid uptake. *Cd36* plays a central role in hepatic steatosis by facilitating long-chain fatty acid transport into hepatocytes, and its upregulation has been consistently associated with MASLD progression and lipid accumulation (Miquilena-Colina et al., 2011). Therefore, the observed reduction in *Cd36* transcript levels suggests its involvement in fatty acid transport when microbial metabolites are administered; this may be associated to the reduction of lipid accumulation into hepatocytes under lipotoxic conditions (Rada et al., 2020). While direct causality cannot be established within the present model, the concordance between reduced lipid accumulation and lower *Cd36* transcript levels suggests that microbiota-derived metabolites may modulate hepatic lipid handling under lipotoxic conditions. These results are consistent with current evidence indicating that gut microbiota-derived metabolites contribute to the link between dietary substrates and host cell metabolic regulation (Tilg et al., 2016; Tripathi et al., 2018).

Overall, the study is subject to inherent limitations, including the simplified microbial composition relative to native gut ecosystems and the constraints of an *in vitro* hepatocyte model, which does not fully recapitulate systemic host responses. Likewise, the reductionist nature of the model allows a more detailed understanding of how the microbial interactions modulate the whole community at strain level and how the microbial metabolic profile influences the microbial community. However, the specific metabolites responsible for the observed effect on host cells were not identified based on the utilized approach of the study. Although the reductionist reconstructed microbiota model used here allows controlled investigation of strain-level interactions and FOS-driven metabolic drivers, it does not reproduce the taxonomic complexity, donor-specific variability, spatial organization, intestinal barrier function, or host-mediated processes occurring *in vivo*. Future studies should therefore evaluate these findings using more complex and physiologically relevant *in vitro* models, including donor-derived microbiota-based approaches such as RapidAIM, dynamic gut fermentation systems, and integrated gut-liver platforms to validate the mechanistic insights.

In conclusion, these findings suggest that FOS-driven metabolism within a defined *Lactobacillus* reconstructed microbiota may contribute to influence host metabolic responses by shaping microbial metabolic profiles and may be associated with reduced lipid accumulation in steatotic HepG2 cells. However, the observed effects should be interpreted as metabolite-associated responses in a reductionist model and require validation in more complex gut-liver systems.

## Data Availability

The original contributions presented in the study are included in the article/Supplementary material, further inquiries can be directed to the corresponding author.

## Supplementary material

Supplementary Figure S1 Expression of genes involved in sucrose/FOS metabolism in *L. plantarum* PBS067 during growth as single strain on 2% FOS DP ~ 10 at 24 h. Gene expression was assessed by RT-qPCR and normalized to strain-specific 16S rRNA reference genes. Data are reported as 2^(−ΔΔCt)^ ± standard deviation.

Supplementary Figure S2 Expression of probiotic genes involved in sucrose/FOS metabolism in the probiotic consortium during growth on 2% FOS DP ~ 10 at 24 h. Gene expression was assessed by RT-qPCR and normalized to strain-specific 16S rRNA reference genes. Data are reported as 2^(−ΔΔCt)^ ± standard deviation.

Supplementary Figure S3 Chromatogram profiles of bacterial metabolites by GC-MSD after upon on 1% glucose. Metabolites produced by **(A)** probiotic consortium, **(B)** minimal core, and **(C)** the whole community.

Supplementary Figure S4 **(A)** Viability of HepG2 cells treated with 0.25 mM palmitic acid for 24 h. Values are represented as mean value percentage of vitality ± standard error. **(B)** Lipid accumulation in HepG2 cells treated with 0.25 mM palmitic acid for 24 h. Values are represented as mean red intensity percentage ± standard error. **(C)** Light microscopy images at 40 × magnification (scale bar: 10 μm) of non-treated cells and cells treated for 24 h with 0.25 mM palmitic acid. **(D)** Expression of genes potentially involved in MASLD (*SREB, ACC, Fasn, PPAR*α, *HMGCR, Cd36*) in HepG2 cells treated with 0.25 mM palmitic acid for 24 h. Values are represented as mean fold respect to control ± standard error. Statistical differences were calculated using Student's *t*-test: ^*^*p*-value < 0.05, ^**^*p*-value < 0.01, ^***^*p*-value < 0.001, ^****^*p*-value < 0.0001.

Supplementary Table S1 Species-specific primers used in this study.

Supplementary Table S2 Primers used in this study to analyse FOS gene expression.

Supplementary Table S3 Primers used in this study to analyse HepG2 gene expression.

Supplementary Table S4 Probiotics genes identified in this study.
