# Supplementary material for: Fructooligosaccharides-driven metabolism of a defined Lactobacillus probiotic consortium modulates hepatic lipid accumulation in a reductionist in vitro gut microbiota–liver interaction model
Source: Front Microbiol. 2026 Sep 9;17:1866842. doi: 10.3389/fmicb.2026.1866842 (PMC13599725; doi:10.3389/fmicb.2026.1866842)
Supplement: Supplementary file 1 [file Table_1.docx]

**Table S1** Species-specific primers used in this study.

| **Strain** | **Primer code** | **Sequence** | **Target** | **Reference** |
| --- | --- | --- | --- | --- |
| *L. plantarum* | LP_16S_F | GCATCATGATTTACATTTGAGTG | 16S | This study |
|  | LP_16S_R | CTCGGACCATGCGGTCCAAG |  |  |
| *L. acidophilus* | LA_16S_F | CGGTGATGACGTTGGGAACG | 16S | This study |
|  | LA_16S_R | ATAAGCTGATCATGCGATCT |  |  |
| *L. reuteri* | LR_16S_F | TGATTGACGATGGATCACCAGTG | 16S | This study |
|  | LR_16S_485R | GGGTTGGATACCGTCACTGCG |  |  |
| *B. cellulosilyticus* | BAC_16S_F | GGTAGTCCACACAGTAAACGATGAA | 16S | Dejean et al., 2020 |
|  | BAC_16S_R | CCCGTCAATTCCTTTGAGTTTC |  |  |
| *C. symbiosum* | CS_16S_F1 | ACACGTGCTACAATGGCGTA | 16S | This study |
|  | CS_16S_R1 | TCACTGACTTCGGGCGTTAC |  |  |
| *F. plautii* | CO_16S_F1 | CCTTCGGGGAAAGTGGAGAC | 16S | This study |
|  | CO_16S_R1 | GCCTCCCTCTGTTAACCACC |  |  |
| *E. coli* | EC_F | CATGCCGCGTGTATGAAGAA | 16S | Huijsdens et al., 2002 |
|  | EC_R | CGGGTAACGTCAATGAGCAAA |  |  |

**Table S2** Primers used in this study to analyse FOS gene expression.

| **Gene** | **Primer code** | **Sequence** | **Reference** |
| --- | --- | --- | --- |
| LP_SacK1 | LP_SacK1_F | AGTCGCTTCAACATCCCACT | This study |
|  | LP_Sack1_R | CTTGAAATCGTCCCCAGGTA |  |
| LP_SacA | LP_SacA_F | CCAGATACGCCGTACGACTC | This study |
|  | LP_SacA_R | ACCGATTAGCGCGTAGTAGC |  |
| LR_SacK1 | LR_Sack1_F | GCAATTTTGTTGGCACAATG | This study |
|  | LR_Sack1_R | AAGCGACCATCTTGAATTGC |  |
| LA_ScrB | LA_ScrB_F | TTTGCAAGCACAAGCAGCAA | This study |
|  | LA_ScrB_R | ATGTTCGGTGATGTGGTCAGGATT |  |

**Table S3** Primers used in this study to analyse HepG2 gene expression.

| **Gene** | **Primer** | **Sequence** |
| --- | --- | --- |
| Pol2 | Fw | AGGAGCAAAGCCTGGTGTT |
|  | Rv | ACCCAAAGCTGCCAGAAGT |
| SREBP | Fw | GAGCTCAAGGATCTGGTGGT |
|  | Rv | CCGACACCAGATCCTTCAGA |
| ACC | Fw | CAACTTTGTGCCCACGGTTA |
|  | Rv | TTTGTCAGGAAGAGGCGGAT |
| Fasn | Fw | CCCTCATCTCCCCACTCATC |
|  | Rv | CAGCGTCTTCCACACTATGC |
| PPARα | Fw | ATCGGCGAGGATAGTTCTGG |
|  | Rv | CCTTGTCCCCGCAGATTCTA |
| HMGCR | Fw | GACCTTTCCAGAGCAAGCAC |
|  | Rv | CCCCTGACATGGTACCAACT |
| Cd36 | Fw | TGGTACAGATGCAGCCTCAT |
|  | Rv | AGGCCTTGGATGGAAGAACA |

**Table S4** Probiotics genes identified in this study.
